# Confident methods for the evaluation of the hydrogen content in nanoporous carbon microfibers

**DOI:** 10.1186/1556-276X-7-588

**Published:** 2012-10-24

**Authors:** Mario Culebras, Antonio Madroñero, Andres Cantarero, José Maria Amo, Concepción Domingo, Antonio López

**Affiliations:** 1 Materials Science Institute, University of Valencia, 46071 Valencia, PO Box 22085, Spain; 2 CENIM, , Av. Gregorio del Amo 8, 28040 Madrid, Spain; 3 Instituto de Estructura de la Materia (CSIC), , C/ Serrano 121, 28006 Madrid, Spain; 4 CIEMAT, , 04200 Tabernas, Almeria, Spain

**Keywords:** Raman dispersion, Chemical vapor deposition, Nanoporous materials

## Abstract

Nanoporous carbon microfibers were grown by chemical vapor deposition in the vapor-liquid solid mode using different fluid hydrocarbons as precursors in different proportions. The as-grown samples were further treated in argon and hydrogen atmospheres at different pressure conditions and annealed at several temperatures in order to deduce the best conditions for the incorporation and re-incorporation of hydrogen into the microfibers through the nanopores. Since there are some discrepancies in the results on the hydrogen content obtained under vacuum conditions, in this work, we have measured the hydrogen content in the microfibers using several analytical methods in ambient conditions: surface tension, mass density, and Raman measurements. A discussion on the validity of the results obtained through the correlation between them is the purpose of the present work.

## Background

Hydrogen is known to be the most common element in the Milky Way, and it represents 74% in content, followed by helium (24%), oxygen (1%), and carbon (0.4%) [1]. It is found in a large amount of chemical compounds, particularly in carbon-rich and organic materials. Atomic hydrogen is unstable, and it is usually found in combination with other elements (hydrocarbons, polymers, water, etc.) or as a diatomic molecule. Hydrogen is used, as least in prototypes, in fuel cells, which is a very important issue in energy storage [2]. Another interesting application is thermoelectricity; the electrical conductivity and the Seebeck coefficient can be engineered by changing the hydrogen content [3].

On average, the storage capacity of hydrogen in carbon nanostructures is of the order of 1.5 wt.%, although the storage capacity can significantly change with the desorption temperature [4] or hydrostatic pressure [5]. For instance, single-walled carbon nanotubes (CNTs) show a hydrogen uptake of 5 to 10 wt.% at 133 K and 40 kPa [6]. It has also been shown that single- or multi-walled CNTs adsorbed a hydrogen amount of 3 to 4 wt.% at room temperature but at 10 MPa [7,8].

An important problem in this research field is to have a confident measurement of the hydrogen content. This is not an easy matter because of the depletion of hydrogen when the fibers are in a vacuum environment [9], and many of the used techniques need vacuum conditions. Techniques such as elastic recoil detection analysis show unsatisfactory sensitivity since it works with the samples placed into a high vacuum chamber [10]. It was also difficult to obtain confident results in the measurement of the hydrogen content by reflection electron energy loss spectroscopy; the measurement error was not lower than 20% [11].

In this work, we study carbon fibers grown by chemical vapor deposition, a method which allows obtaining a good-quality material under a reasonable cost. The vapor growth produces filaments of some centimeters of length and microfibers with a length smaller than 100 *μ*m. The manufacturing process of vapor-grown carbon fibers (VGCFs) has been previously described in the literature [12]. They have been prepared incorporating metallic particles of group VII to the gas flow entering into the reactor. Although VGCFs synthesized with a metallic catalyst have received special attention in many fields because of their controllable structure and attractive mechanical and electrical properties [7,13], one of the most important applications of VGCFs in the near future will be as hydrogen storage materials, mainly in the form of nanowires because of its large surface for hydrogen incorporation. In this work, we have studied the hydrogen content in VGCFs, both filaments and microfibers, restricting the analysis to techniques which do not need vacuum, and try to select the more confident technique for measuring the hydrogen content in porous materials, particularly in carbonaceous specimens.

## Methods

### Crystal growth and sample preparation

The precursors for the growth of samples A to C, and D and E are basically hydrogen, methane, ethane, and benzene. They have been grown in vapor-liquid-solid mode in a furnace at 1,323 K within a quartz ampule. The microfibers were prepared using small iron particles as catalyst. These particles came from minute drops of ferrocene solved in alcohol. Once evaporated, they were placed in a hot hydrogen environment. In this way, they have been turned into minute chips of active metallic iron. The growth temperature of our furnace was 1,323 K in all cases. As a result, minute fiber-type flying seeds (FS) are created. If the catalyst particles are deliberately placed on a substrate, carbonaceous microfilaments or filaments grown on a substrate (FGS) of some centimeters of length are created on the substrate. The description of the raw atmosphere for the sample preparation is given in Table 1. The five samples (A to E) have received further treatments to analyze the incorporation and refilling of hydrogen (producing samples F to I). The raw atmosphere, temperature, and pressure conditions for the fabrication of samples F to I are shown in Table 2.

**Table 1:** Set of samples grown as explained in the text

**Sample**	**Type**	**Raw atmosphere**
A	FGS	70% H_2_ + 30% CH_4_
B	FGS	H_2_ bubbling in C_6_H_6_
C	FGS	70% H_2_ + 15% CH_4_ + 15% C_2_H_4_
D	FS	70% H_2_ +30% CH_4_
E	FS	H_2_ bubbling in C_6_H_6_

Samples A, B, and C are filaments grown on asubstrate and samples D and E flying seed type fibers. FGS, filaments grown on a substrate; FS, flying seed type fibers.

**Table 2:** Samples obtained after further treatment on samples A and B (FGS)

**Sample**	***T***_**A**_ (K)	**Atmosphere**	**P(bar)**	***t***_**A**_**(h)**
F(A)	727	Ar	1	1
G(A)	927	Ar	0.2	2
H(G)	RT	H_2_	200	24
I(H)	RT	H_2_	0.00133	24
J(A)	RT	H_2_	200	24
K(B)	RT	H_2_	200	24

Column 2 shows the annealing temperature, and the last column, the annealing time. RT, room temperature.

#### Thermogravimetric measurements

In order to carry out a rough comparison between the samples, thermogravimetric measurements (TGA) were performed using a TGA system. The samples were kept in Ar atmosphere, and the heating velocity was 2 K/min between 323 and 473 K and 20 K/min between 473 and 1,023 K.

#### Surface energy measurements

In the present study, we used the contact angle measured in a sessile drop test as the way to evaluate the surface area. Following Fowkes [14], the following relationship exists between the contact angle *θ* and the surface energies *γ*_*L*_ and *γ*_*S*_ of the liquid and solid, respectively:

(1) $$\frac{\gamma_{L}(1+\cos\theta )}{2}=\sqrt{\gamma_{S}^{p}\gamma_{L}^{p}}+\sqrt{\gamma_{S}^{d}\gamma_{L}^{d}}\;,$$

where the upper index *p* and *d* are used to distinguish the polar and dispersive components ( $\left(\gamma_{L}=\gamma_{L}^{p}+\gamma_{L}^{d}\right)$; $\left(\gamma_{S}=\gamma_{S}^{p}+\gamma_{S}^{d}\right)$) of the surface energy. By using two liquids, a polar liquid (for instance, glycerol: $\left(\gamma_{L}^{d}=37\right)$ mN/m and $\left(\gamma_{L}^{p}=26.4\right)$ mN/m) and a non-polar liquid (as vaseline oil: $\left(\gamma_{L}^{d}=22.3\right)$ mN/m), we were able to obtain $\left(\gamma_{S}^{p}\right)$ and $\left(\gamma_{S}^{d}\right)$ from the measurements of the contact angles with the VGCFs.

#### Density measurements

In the work of Madroñero et al. [15], it was shown that the external part of the VGCFs is composed of two phases: an amorphous matrix and graphitic platelets. The hydrogen adsorption takes place basically in the amorphous phase because the accumulation of hydrogen is more intensive in defects [16]. According to that, it is reasonable to assume that the relationship between the amount of stored hydrogen and the density should not be very different from the established relation for amorphous hydrogenated films [17]. It can be accepted that the relation between density and hydrogen content follows the Gaussian expression [18]:

(2) $$\rho =1.79-0.4274\;e^{-2{\left[(x-0.46)/0.28\right]}^{2}}$$

where *ρ* is the density in g/cm^3^ and *x* is the hydrogen content in wt.%. As the first step, the mass of each sample was determined. Then, the volume was established using a gas pycnometer with helium. The hydrogen content of each sample has been calculated from Equation 2.

#### Raman measurements

A Raman confocal microscope (Renishaw 2000, Renishaw, Gloucestershire, UK) has been used in the analysis. It was provided with a Leica microscope (Leica, Solms, Germany), a nitrogen-cooled charge couple device, and an air-cooled Ar ion laser (514.5 nm) as excitation source. A ×50 objective has been utilized. The spectra of all the samples have been evaluated by the own software of the system.

## Results and discussion

**Figure 1:**
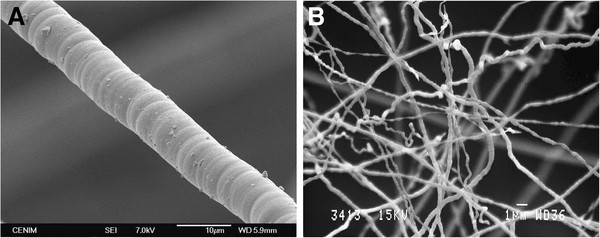
SEM images of a filament grown on a substrate (A) and a microfiber-type flying seed (B).

Figure 1A,B shows two images obtained by scanning electron microscopy (SEM). The fibers observed in these images were fabricated from methane and hydrogen (samples A and D following Table 1): the first image shows a FGS fiber, while the second, a FS fiber.

**Figure 2:**
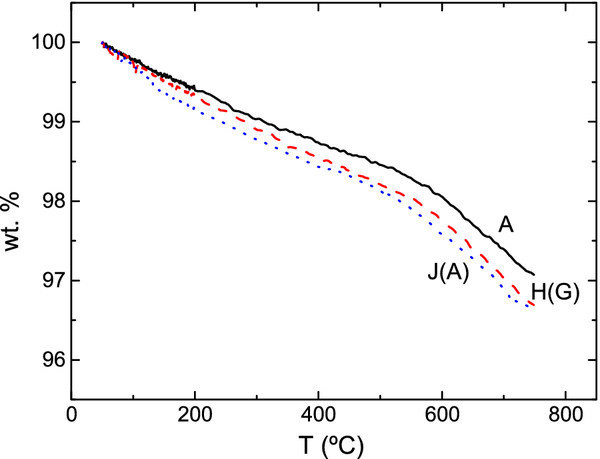
**Thermogravimetric records.** Samples A (solid black line), H(G) (dashed red line), and J(A) (dotted blue line).

Thermogravimetric tests correspond to three representative samples, one as grown (A), a second sample overcharged (J(A)), and a third one recharged after clearing (H(G)). The objective of these tests was to check if some irregularities in the curve of weight loss may suggest complexity in the outgassing mechanism. Figure 2 shows the thermograms of samples A, J(A), and H(G). As can be observed, there is no preferential temperature for the hydrogen taking off in the outgassing process. This fact may suggest a simple process of outgassing without a hydrogen diffusion mechanism [19], which has been widely described in the scientific literature. 

The Raman spectra of carbonaceous materials consist of two main broad peaks known as G -band ( G stands for ‘graphite’) and D -band (proportional to the level of ‘disorder’ or defects) [20]. The G -band is located around 1,575/cm, and it is assigned to a doubly degenerated deformation vibration of the hexagonal ring corresponding to the *E*_2*g*_ mode of graphite with $\left(D_{6h}^{4}\right)$ crystal symmetry. The D -band is located around 1,355/cm, and it is an indication of the crystal size [21]. The existence of the D -band points out the existence of disorder-induced scattering. During the process of carbonization of polymers through thermal treatment at increasing temperatures, the intensity of the D -band decreases (indicating recrystallization). When the D -band disappears completely, the material has turned into a well-ordered graphite [22] material with no defects (obviously, a neglected defect concentration).

Therefore, we may suggest that the variation of the ratio between the intensity of the D  and G peaks indicates an alteration in the graphitic character of the material [23]. For this reason, the relationship between the intensity of the peaks D/G has been generally used to predict the elastic modulus of carbon thin films [24] and CNTs [25]. This relationship points out the alteration of crystalline perfection.

**Figure 3:**
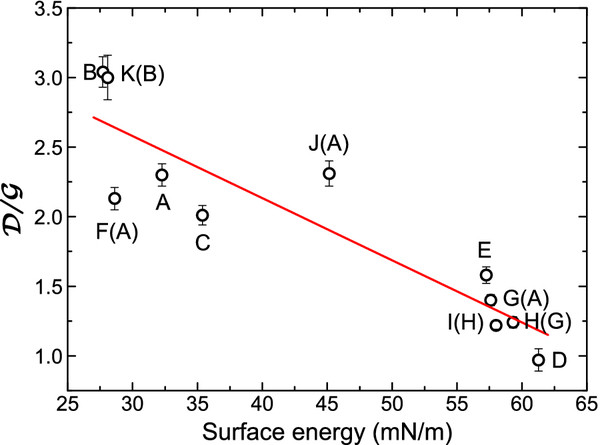
**Correlation between surface tension and the Raman ratio**D/G .

We have correlated the surface energy and density measurements with that of the Raman ratio of the D/G intensities. Figure 3 shows the correlation between the surface energy measure with the sessile drop method as indicated in the previous section with that of the Raman ratio D/G . Although the line is a linear square fit, there is no evidence of a linear correlation, but we can roughly observe an increase of the surface tension when the quantity of defects (the decrease of the D  intensity in the Raman spectra) is decreased. A first point to be noticed in the figure is that the FGS samples have a smaller surface tension (27−35mN/m) than that of the FS samples (57 to 62 mN/m), nearly one half, and a smaller defect concentration. By looking at Table 2, we can realize that the increase in the annealing temperature and the outgassing time on sample A (F(A) and G(A)) increases enormously the surface tension and probably the recrystallization. Annealing sample A (J(A)) in a hydrogen atmosphere and high pressure increases the number of defects and the surface tension. In sample B, however, the annealing with hydrogen at high pressure (K(B)) produces nearly no effect in the surface tension or recrystallization. The same happens for samples H(G) (refilling) and I(H) (annealed in vacuum in a residual hydrogen atmosphere); the surface tension and number of defects is very close to sample G(A).

**Figure 4:**
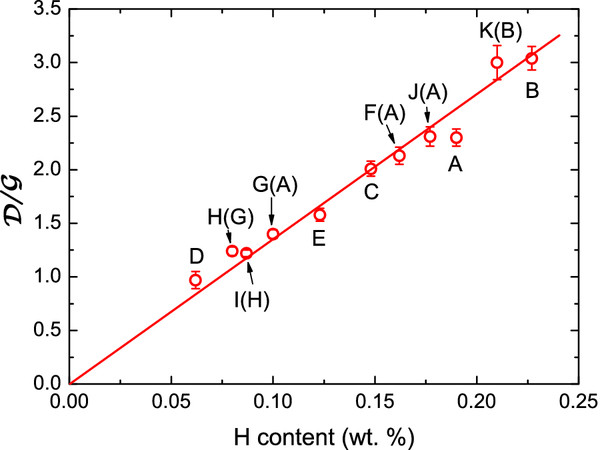
**Correlation between**D/G **and the hydrogen content derived from density measurements.** The line is a linear fit, keeping only the slope as a parameter. Actually, it does not necessarily cross zero (perfect sample with no defects).

In Figure 4, the change of D/G as a function of the hydrogen content derived from the density measurements using Equation 2 is shown. Given the good linear correlation between the ratio D/G and the hydrogen content, we have made a linear fit. Although it crosses zero because we left only the slope as fitting parameter in the linear fit, it may not necessarily cross zero. There is probably, at some point, a saturation effect given a finite value of the Raman ratio for zero hydrogen content since a sample without hydrogen is not necessarily perfect. From the comparison of the results for samples A, B, and C shown in Figure 4, it is observed that the feedstock atmosphere composition have an observable but not very remarkable influence on the hydrogen content in the case of FGS; to a methane atmosphere precursor corresponds a hydrogen content of 0.19%, to a benzene precursor corresponds a hydrogen content of 0.22%, and to methane-ethylene, 0.15% of hydrogen. In FS fiber samples (D and E), the hydrogen content is smaller than in the FGS. In the case of sample G(A), the desorption is more pronounced because besides the temperature, the vacuum per se produces an outtake of hydrogen in this type of fibers [26]. The small difference between the hydrogen content of samples A and J(A) suggests that the refilling from a high-pressure atmosphere of hydrogen is not effective, and the sample to be recharged was not activated for hydrogen adsorption [7]. The same conclusion is valid for the comparison between K(B) and B. The more unexpected change is that observed in the variation from G(A) to H(G). The question is that G(A) was submitted to an annealing with a temperature higher than 500°C. It is well established that spangles of graphene after an annealing around 500°C releases all the fluctuant hydrogen, and the C-H bonds resting in the final material are very stable because they are *sp*^3^ bonds [27]. 

## Conclusions

From the analysis and comparison of the different techniques used to measure the hydrogen content in the carbonaceous materials, we can conclude that the density and Raman measurements are the most confident techniques since there is a clear linear correlation between the hydrogen content extracted from the density measurements and the D/G ratio of the Raman peaks. In any of these techniques, vacuum is needed for the measurements, and we can ignore the discussion or the evaluation on the effect of the vacuum conditions on the final results. From the results, we can also conclude that the samples with more hydrogen content are those grown bubbling hydrogen in benzene: B compared with C and A (FGS), and E compared to D (FS).

## Competing interests

The authors declare that they have no competing interest.

## Authors’ contributions

AM grew the carbon fibers. MC did the thermal treatments. JMA carried out the density and surface tension measurements. CD performed the Raman measurements. AL performed the TGA measurements. Finally, AC made the Raman analysis and curve fits. All authors read and approved the final manuscript.
